# Enteral Nutrition and Acute Pancreatitis: A Review

**DOI:** 10.1155/2011/857949

**Published:** 2010-08-03

**Authors:** B. W. M. Spanier, M. J. Bruno, E. M. H. Mathus-Vliegen

**Affiliations:** ^1^Department of Gastroenterology and Hepatology, Rijnstate Hospital, P.O. Box 9555, 6800 TA Arnhem, The Netherlands; ^2^Department of Gastroenterology and Hepatology, Erasmus MC University Medical Center, 3000 CA Rotterdam, The Netherlands; ^3^Department of Gastroenterology and Hepatology, Academic Medical Center, University of Amsterdam, 1105 AZ Amsterdam, The Netherlands

## Abstract

*Introduction*. In patients with acute pancreatitis (AP), nutritional support is required if normal food cannot be tolerated within several days. Enteral nutrition is preferred over parenteral nutrition. We reviewed the literature about enteral nutrition in AP. *Methods*. A MEDLINE search of the English language literature between 1999–2009. *Results*. Nasogastric tube feeding appears to be safe and well tolerated in the majority of patients with severe AP, rendering the concept of pancreatic rest less probable. Enteral nutrition has a beneficial influence on the outcome of AP and should probably be initiated as early as possible (within 48 hours). Supplementation of enteral formulas with glutamine or prebiotics and probiotics cannot routinely be recommended. *Conclusions*. Nutrition therapy in patients with AP emerged from supportive adjunctive therapy to a proactive primary intervention. Large multicentre studies are needed to confirm the safety and effectiveness of nasogastric feeding and to investigate the role of early nutrition support.

## 1. Introduction

Acute pancreatitis (AP) ranges from a mild and self-limiting disease (80%), which usually resolves spontaneously within days, to a rapidly progressive fulminant illness with significant morbidity and mortality [1, 2]. The two most common etiological factors, representing more than 80% of cases, are gallstones and alcohol abuse [1, 3]. 

The clinical course of an attack of AP varies from a short period of hospitalization with supportive care to prolonged hospitalization and admittance to an Intensive Care Unit (ICU) because of the development of systemic inflammatory response syndrome (SIRS), multiorgan failure (MOF), and septic complications. Overall, in about 15% to 20% of patients, AP progresses to a severe illness with a prolonged disease course. These severely ill patients may develop organ failure and/or local complications such as pancreatic necrosis. In patients with necrotizing pancreatitis, the mortality is close to 17%, with a mortality of 12% in the case of sterile necrosis and up to 30% in infected necrosis [1]. 

Usually, the initial treatment of AP consists of a nil per os (NPO) regimen and the administration of analgesics and ample intravenous fluids [1, 2, 4]. The rationale for a period without food intake is the assumption that pancreatic stimulation by enteral feeding may aggravate pancreatic inflammation. The validity of this concept of “pancreatic rest” is heavily debated [5–7]. Moreover, many patients are anorectic and may suffer increasing pain sensations when eating and ileus-related nausea and vomiting. The resumption of oral feeding depends on the improvement of abdominal pain, absence of nausea and vomiting, and return of appetite. Nutritional support is required in those patients who cannot tolerate normal food within several days [1, 4, 8, 9]. 

To date, there is a substantial scientific proof that enteral feeding is superior to total parenteral nutrition (TPN) [5, 6, 10–15]. The beneficial effects of enteral feeding on mucosal integrity and the prevention of bacterial overgrowth may well explain the superiority of enteral feeding over TPN [16, 17]. Enteral feeding significantly reduces the risk of infections, lowers the need for surgical interventions, and reduces the length of hospital stay [5, 6, 10–12, 15, 18]. Recently, Petrov and coworkers concluded in their meta-analysis that mortality is significantly reduced when patients with a predicted severe AP are fed enterally [14]. Importantly, this reduction of mortality in patients with a severe AP may also be related to the timing of the start of nutrition, within 48 hours after admission [13]. Whenever enteral nutrition is initiated, issues such as the ideal composition, timing, and route of delivery should be considered, as they may all impact on the outcome of AP. Patients who are unable to tolerate enteral nutrition need to be managed with TPN until such time that they can tolerate enteral feeding [9]. In this review an update is given about several aspects of enteral nutrition in mild and severe AP.

## 2. Pancreatic Rest and Pancreatic Secretion

Efforts to keep up with the increased energy demands in the case of AP are thwarted by the adage to put the pancreas at rest and the avoidance of pancreatic stimulation via gut luminal nutrition. As mentioned in the introduction, this adage merits reconsideration. The concept of “putting the pancreatic to rest” assumes that pancreatic rest promotes healing, decreases pain, and reduces secretion and leakage of pancreatic juices in pancreas parenchyma and peripancreatic tissue [19]. The concept of pancreatic rest originates from the classic work of Ragins et al. based on a canine model [20]. They demonstrated that jejunal feeding did not stimulate pancreatic secretion as opposed to intragastric or intraduodenal feeding. However, this concept of “putting the pancreas to rest” disregards the persistence of basal pancreatic exocrine secretion. Of the three components of pancreas secretion (protein enzymes, fluid volume, and bicarbonate), protein enzyme output is responsible for autodigestion of the gland and perpetuation of the inflammatory process [19]. Suppression of protein enzyme output alone with continued bicarbonate and fluid volume output may therefore be adequate in putting the pancreas to rest.

### 2.1. Physiology of Pancreatic Secretion

Basal enzyme secretion is 20% of maximal enzyme secretion and is regulated by cholinergic and cholocystokinin (CCK)-mediated mechanisms. Feeding by mouth increases pancreas secretion by involving three levels of stimulation via three interrelated phases: the cephalic, gastric, and intestinal phase [21, 22]. The cephalic phase is mediated through direct cholinergic stimulation by the vagus nerve on pancreatic acinar cells. The vagus also acts indirectly by stimulating gastrin release from the antrum and vasoactive intestinal peptide (VIP) release from the small intestine. The gastric phase of pancreatic secretion has not been fully elucidated. Gastrin affects pancreatic secretion by two mechanisms: gastric acid secretion, resulting in secretin secretion when a low pH reaches the duodenum and a direct effect of gastrin on acinar cells to produce an enzyme-rich secretion. The intestinal phase accounts for the majority of postprandial exocrine pancreatic secretory output and is orchestrated by multiple mediators: vagus nerve, CCK and VIP, a secretin-like hormone, and cholinergic enteropancreatic reflexes. 

Human pancreatic enzyme output reaches maximal rates following a mixed meal of 20 kcal/kg body weight [23, 24]. The duration of the response increases with greater caloric load. The pancreatic response is also influenced by the physical properties of the meal: mixed solid-liquid meals induce a higher response than liquid or homogenized meals with a similar energy content. In both instances, the rate of gastric emptying and thus duodenal delivery of nutrients are the key factors which determine the duration of the pancreatic secretion. Proportion of fat, carbohydrate, and protein contents within a meal also influence the duration and enzyme composition of the pancreatic response in humans.

### 2.2. Pancreatic Secretion with Total Enteral Nutrition

Recent human studies show that all common forms of EN to some extent stimulate the pancreas and only parenteral nutrition avoids pancreatic stimulation [25, 26]. Considerable evidence exists that the degree to which the pancreas is stimulated by enteral nutrition (EN) is determined by the site in the gastrointestinal tract at which feedings are infused. Feeding infused into the jejunum beyond the ligament of Treitz may bypass the cephalic, gastric, and intestinal phase of stimulation of pancreatic secretion, is less likely to stimulate CCK and secretin, and may stimulate inhibiting polypeptides [27–29]. It has been demonstrated in human studies during jejunal feeding, that pancreatic enzyme output increased significantly over basal levels when it was delivered at the ligament of Treitz, whereas there was no significant increase during more distal jejunal feeding, 60 cm beyond the ligament of Treitz [30]. A more recent study in healthy volunteers showed that EN can be given without stimulating pancreatic trypsin secretion provided that it is delivered into the mid-distal jejunum [31]. Feeding from 20 to 120 cm beyond the ligament of Treitz had no stimulatory effect.

Also the composition of the infused feeds is important. There is considerable evidence to support an added benefit of elemental formulae for putting the pancreas to rest compared to standard formulae with intact protein or blenderized diets [19]. Elemental diets cause less stimulation than standard formulas, because of their low fat content, the presence of free aminoacids instead of intact proteins which bind to free trypsin in the gut, causing trypsin levels to fall, and less acid production from the stomach.

### 2.3. Outcomes of Pancreatic Rest

Whether pancreatic rest has a role to play in patients with severe AP is still uncertain, as no well-powered randomized, prospective studies have been carried out to address this specific question [6]. Whether pancreatic rest is at all needed is questioned by the results of several studies comparing nasogastric with nasojejunal feeding in severe AP, as nastrogastric feeding appears to be safe and well tolerated [32, 33]. 

Putting the pancreas to rest is based on the assumption that the inflamed and/or necrotic pancreas is still a secretor of activated enzymes once stimulated. Animal studies have shown that pancreatic exocrine secretion in experimental AP in response to CCK stimulation is suppressed [34]. A small prospective study showed pancreatic exocrine insufficiency to be common in patients recovering from severe AP; its severity correlated with the extent of pancreatic necrosis [35]. Another study demonstrated that trypsin secretion in AP patients, especially with necrotizing AP, is significantly suppressed compared to healthy individuals. However, despite these low rates of luminal secretion, the rate of appearance of newly synthesized trypsin was unchanged [36]. Thus, a more likely alternative explanation for the absence of exacerbation of the disease during EN is that the pancreas becomes less responsive to EN stimulation during an attack of AP and that the secretory response to EN is suppressed to basal rates [34, 37]. However, there is still some doubt whether the pancreatic secretion is fully suppressed. Overall, the concept of pancreatic rest seems to be less probable.

## 3. Outcomes of Nutritional Support

Nutritional therapy in the past has been governed by the principle that the gut should be put at rest with avoidance of any stimulation of pancreatic exocrine secretion. These concepts should now be replaced by the principle that pancreatic stimulation should be reduced to basal rates, but that gut integrity should be maintained and that the stress response should be contained to reduce the likelihood of multiorgan failure, nosocomial infections, and mortality [38]. 

The question remains if nutritional support is beneficial for the outcome of AP. Powell et al. published the only randomized controlled trial comparing EN with no nutritional support and studied the effect of early EN on the markers of inflammatory response in severe AP [39]. Nutrition therapy provided by the enteral route did not have a more favorable impact on patient outcome than standard therapy as no differences were found between the two groups with respect to overall complications, length of hospital stay, or the time to resume an oral diet. Serum markers of inflammation appeared to be lower in the group receiving EN compared with those randomized to standard therapy, but none of the differences was statistically significant. The findings of this study suggest that low-caloric EN does not modify the inflammatory response in severe AP, but limits in the design of the study should be mentioned: only 21% of the caloric requirements were infused in the EN group, the study duration was only 4 days, and small numbers of patients were recruited. 

As already mentioned, the pancreas is in a state of unresponsiveness during an attack of AP and the secretion of pancreatic juice and trypsin is reduced during AP [34, 37]. Eckerwall et al. investigated the role of immediate oral feeding versus fasting in 60 patients with AP [40]. All patients received initial aggressive fluid resuscitation to maintain intravascular circulatory volume, microcirculation, and renal function, thereby minimizing the extent of ischemia and reperfusion injury. Compared to the fasting patients, the orally fed group had a significantly shorter period of intravenous fluids, less days of fasting, and a 2-day earlier introduction of solid foods, with no differences in blood chemistry, gastrointestinal symptoms, complications, and interventions. The orally fed group had a significant 2-day shorter length of hospital stay without differences in recurrent attacks of pancreatitis in a follow-up of 3 months.

## 4. Modifications of Enteral Nutrition

### 4.1. Standard Composition: Elemental, Semielemental, or Polymeric Formulas

Few studies to date compare the results of feeding elemental, semielemental, and polymeric diets to patients with AP [7, 15]. Elemental formula are completely predigested and consist of aminoacids, simple sugars, and enough fat to prevent essential fatty acid deficiency. Semielemental formulas required less digestion than polymeric foods and contain peptides of varying chain length, simple sugars, glucose polymers, or starch and fat primarily as medium chain triglycerides. Polymeric feeds contain nonhydrolyzed proteins, complex carbohydrates, and long chain triglycerides. Based on the assumption that elemental and semielemental formulas cause less pancreatic stimulation than standard formulas, most EN studies have used an elemental or a semielemental formula. It would seem that the location of the enteral tube is just as important as the type of enteral formulas in stimulating pancreatic secretion. Few data exist on the use of standard enteral formula in such patients. Both Windsor et al. and Pupelis et al. have shown that polymeric formula can be safely fed through jejunal tubes in AP patients [17, 41]. In a longitudinal study by Makola et al., 126 patients received standard formula via a jejunal tube which was inserted through a percutaneous endoscopic gastrostomy (PEG-J) [42]. The standard enteral formula resulted in both a significant decrease of the median CT severity index and increase of serum albumin compared from baseline to the time of tube removal. Few studies have defined the benefits of semielemental versus polymeric formulas in severe AP. In 1989, Cravo et al. found a similar tolerance in 102 patients with AP given semielemental versus polymeric formulas [43]. Tiengou et al. compared in a randomized trial semielemental and polymeric formulas in 30 AP patients [44]. Both formulas were well tolerated and well absorbed, but the semielemental group had less weight loss and a shorter length of hospital stay compared with the polymeric group. Recently, Petrov et al. conclude from their adjusted meta-analysis that the use of polymeric, compared with (semi)elemental formulation, does not lead to a significantly higher risk of feeding intolerance, infectious complications, or death in AP patients [45]. It should be remembered that (semi)elemental feeds are sevenfold as expensive as polymeric feeds. In summary, the evidence base to just use (semi) elemental formulas becomes less clear.

### 4.2. Use of Supplements in Enteral Nutrition

Although the use of glutamine supplementation, immunonutrition and prebiotics, and/or probiotics is conceptually sound and attractive, their use is not supported by large-scale studies [15, 46, 47].

Two studies evaluated the use of immune-enhancing formulas, containing glutamine, arginine and fibers or glutamine, arginine, *ω*-3 fatty acids, vitamins, and micronutrients [48, 49]. Hallay et al. compared the effect of a glutamine-rich with a nonglutamine-rich enteral formula on immunologic parameters in 16 patients with AP [48]. The recovery of immunological parameters was better and the time of disease recovery was shorter in the glutamine-treated group. Pearce et al. supplemented in a randomised controlled trial arginine, glutamine, *ω*-3 fatty acids, and antioxidants in 31 patients with severe AP [49]. Surprisingly, an increase in CRP was found in the study group compared with the control group. No significant difference in the length of hospital stay was observed. Although a lower incidence of pneumonia and MOF, and shorter length of ICU- and hospital stay was observed in the immunonutrition group, none of these differences reached statistical significance.

Lasztity et al. randomly administered *ω*-3 fatty acids enterally to 28 patients with moderately severe AP [50]. Supplementation significantly lowered the length of hospital stay and the duration of nutritional therapy, without a significant decrease in overall complication rate. 

Karakan et al. performed the only study to look at a possible role of prebiotics in the attenuation of the severity of AP [51]. They found a significant reduction in hospital stay and duration of the acute phase response in patients receiving prebiotics compared with controls. The study comprised only 30 patients and needs confirmation in larger series.

 Probiotics might prevent infectious complications by reducing small-bowel bacterial overgrowth, restoring gastrointestinal barrier function, and modulating the immune system [52–54]. Oláh et al. demonstrated that *Lactobacillus plantarum* (probiotic) in conjunction with oat fiber (prebiotic) was successful in reducing septic complications (4.5%) versus control (30%) in patients with AP, suggesting that the probiotic therapy enhances the effect of EN in reducing infectious morbidity, however, without a difference in length of hospital stay [55]. Oláh et al. also studied four different prebiotics and probiotics, contained in Synbiotic 2000, and found a decrease in inflammatory response and multiorgan failure in the presence of severe AP [56]. Besselink et al. performed the only large-scale multicenter randomized trial in which 298 patients with a predicted severe AP were randomly assigned to receive a multispecies (Lactobacilli and Bifidobacterium) probiotic preparation or placebo administered enterally [57]. There was no difference in the rate of infectious complications; however, in the probiotic group, the incidence of MOF and the mortality (16% versus 6%) was significantly higher [58]. Nine patients in the probiotics group developed bowel ischaemia, but none in the placebo group. The pathophysiological mechanism that explains why bowel ischaemia developed in patients having had probiotics is unclear, but based on these unexpected study results the use of probiotic prophylaxis in patients with predicted severe AP is highly discouraged. Petrov et al. conclude in their systematic review that supplementation of EN with immunonutrition or probiotics does not significantly improve clinically outcomes and their use is not recommended [45]. Fibre-enriched formulation may be safely administered, but an adequately powered RCT is warranted.

In conclusion, specific supplements added to EN such as arginine, glutamine, *ω*-3 polyunsaturated fatty acids, and prebiotics may be associated with a positive impact on outcome, but studies are too few and underpowered to make strong treatment recommendations [15, 47]. Probiotics should not be administered routinely in patients with predicted severe AP.

## 5. Timing of Enteral Support

The precise timing for initiating enteral support has not been specifically addressed in the pancreatitis population but has been studied to a large extent in the critically ill population. 

 The delivery of EN to critically ill patients early upon admission to the ICU alters physiology in a way that down regulates systemic immunity, reduces overall oxidative stress, and improves patient outcome [59]. Early EN started prior to 48 hours from admission in critically ill patients is associated with a significant 24% reduction in infectious complications and a 32% reduction in mortality compared with delayed feedings started after that point time [59, 60]. 

Marik and Zaloga found in their meta-analysis that “early” EN (within 36 hours) versus delayed EN (after 36 hours) delayed infectious complications and reduced the length of hospital stay in head injury, trauma, burns, postoperative and medical ICU patients [61]. However, caution must be exercised when making inferences about patients with pancreatitis based on information that is gathered from the critically ill. 

 Recently, Petrov et al. conducted a systematic review of randomized controlled trials on the effect of EN versus TPN in patients with mild and severe AP with regard to the timing of nutrition support [13]. EN started within 48 hours of admission resulted in a significant reduction in multiorgan failure, pancreatic infectious complications, and mortality. These significant differences between EN versus TPN faded away when nutrition support started after 48 hours of admission. So EN started within 48 hours of admission may be beneficial and a randomized controlled trial, which has been started, may give a more definite answer.

## 6. Route of Enteral Nutrition Support and Tolerance of Enteral Feeding

Per oral ingestion of nutrients is often hampered by abdominal pain with food aversion, nausea, vomiting, gastric atony, and paralytic ileus or by partial duodenal obstruction from pancreatic gland enlargement [19]. The application of early EN may be limited by the severity of the pancreatitis attack and the occurrence of ileus.

 Traditionally, AP management involved fasting the patient until resolution of symptoms. Recent work has suggested that EN via a jejunal tube is safe and may increase antioxidant activity and reduce the acute phase response and the magnitude of the inflammatory response [17]. 

Most of the feeding tubes are placed as nasojejunal tubes using an endoscopic or a radiologic procedure. Alternatively, especially if the expected period of feeding is 4–6 weeks or more, laparoscopic or radiologic jejunal feeding tubes can be placed. Tolerance is defined by the provision of adequate feeding without ill effects. Tolerance is primary determined by the balance between feeding into the gastrointestinal tract which can be in a state of partial ileus and providing enteral nutrients while causing only minimal stimulation of pancreatic exocrine secretion. Therefore, patients with nasointestinal tubes placed at or below the ligament of Treitz should be monitored very closely for evidence of tube migration as well as evidence of intolerance such as high residual volume, nausea and vomiting, diarrhea, or aspiration of feeding formula. A wide range of tolerance to EN exists irrespective of known influences such as mode (continuous or bolus) and level of infusion within the gastrointestinal tract (gastric versus postpyloric). In patients operated on for complications of AP, continuous infusion appeared to be safer and reduced the stimulation of the pancreas better than bolus infusion [62]. However, insufficient data do not allow a determination of whether continuous or bolus infusion is superior.

After a feasibility study, Eatock et al. performed a randomized controlled study of early nasogastric versus nasojejunal feeding in severe AP [63, 64]. They discovered a surprising tolerance to nasogastric feeding and recommended that nasogastric feeding should be considered a therapeutic option because of its simplicity, obviating the need for endoscopic, radiologic procedures. Eatock's study, however, had several limitations, one of them being the failure to fluoroscopically confirm that the nasojejunal tubes were appropriately positioned in the jejunum. There is no indication whether the nasojejunal tubes were placed distal enough (at least 60 cm from the ligament of Treitz) to avoid gastric and pancreatic stimulation. The failure to find a difference may have been related to continued gastric and duodenal stimulation occurring in both groups of patients. Similar findings from randomized studies were reported by Kumar et al. (nasogastric versus nasojejunal) and Eckerwall et al. (nasogastric versus TPN) [65, 66]. 

Jiang et al. included the 3 RCTs, involving 131 patients, in a meta-analysis [32]. The primary outcome of effectiveness was overall mortality, secondary outcomes of effectiveness were hospital stay, complications and their management. Outcome measure of safety was the occurrence of pain on refeeding and adverse events related to nasogastric EN. The comparator intervention was early EN through a nasogastric tube, the control intervention was one of the conventional pancreatic-rest nutritional support routes of total parenteral or intrajejunal feeding. The meta-analysis showed no significant differences in mortality rate between nasogastric and conventional routes (nasojejunal and parenteral feeding). Also, other outcomes were not different such as length of hospital stay, infectious complications, multiorgan failure, rate of admissions to the ICU, or conversion to surgery. Also, the recurrence of pain on refeeding and adverse events associated with nutrition were similar. 

Petrov et al. performed an extended systematic review which included the 3 RCTs included in the Jiang meta-analysis and the study of Eatock [33]. They also concluded that nasogastric feeding appeared to be safe and well tolerated in the majority (79%) of patients. The aggregated data from the two RCTs comparing nasogastric to nasojejunal feeding showed no statistically significant difference in mortality and tolerance. Both meta-analyses conclude that a well-powered randomized trial on nasogastric versus nasojejunal feeding is indicated to provide a more firm and conclusive evidence to recommend nasogastric feeding as routine clinical practice in patient with acute pancreatitis.

## 7. Timing and Nutrient Composition of Oral Support

Data about when to resume oral feeding in patients with acute pancreatitis or the optimal nutrient composition are scare [40, 67, 68]. The usual criteria to initiate oral feeding are (1) absence of abdominal pain, (2) absence of nausea and vomiting, and return of appetite, and (3) absence of complications. It is possible that the recurrence of pain during the reintroduction of the oral diet is related to ingestion of larger volumes rather than to ongoing or renewed intrapancreatic release of enzymes [64]. Usually, patients are refed small amounts of food frequently during the day and the total number of daily calories is gradually increased over a three- to six-day period [69]. Therefore, feeding is often begun using a clear liquid, a diet for the first 24 hours. If tolerated the diet is advanced to soft low-fat diet over the next 24 hour and then to a low fat solid diet. No clinical trials evaluating these routines are available. A low-fat diet is advised when oral intake is resumed in patients recovering from AP. This is based on the observation that intraduodenal lipids increase volume, bicarbonate, trypsin, and amylase output in volunteers [70]. Besides the presumed stimulation of pancreas exocrine secretion by fat, there might be another reason to postpone fat intake. Pancreatic lipase is less stable than other pancreatic enzymes against acid denaturation and destruction by pepsin and pancreatic proteases, in particularly by chymotrypsin present in chime. This may render lipid digestion more vulnerable in pathologic conditions [71, 72]. Trypsin is not inactivated by acid but only by pepsin. 

Tolerance to advancement to oral diets was evaluated in 274 patients at the point at which abdominal pain had resolved and ileus had subsided [73]. Sixty patients (21.9%) experienced pain relapse and 47 of these 60 subjects pain relapsed within 48 hours of commencement of oral feeding. No pain relapse or pain occurred in those patients randomized to jejunal tube feedings, started a median of 7 days after the onset of symptoms [74]. However, in 4 of the 15 patients (27%) randomized to oral bolus feedings after a median of 5 days after the onset of symptoms, pain on refeeding was associated with longer duration of initial pain and a higher severity index on CT. Lévy et al. came to the same conclusion in a large number of 116 patients [69]. According to the Ranson score ≥3, 35% had a severe AP, and according to the Balthazar CT score >D, this was the case in 42% of patients. Twenty-one per cent of patients had a relapse of pain. The risk of pain relapse increased if serum lipase was greater than three times normal the day before the start of feeding, and was higher in patients who had a longer duration of pain (11 days versus 6 days) and in patients who had a worse CT score (Balthazar score greater than D). The exacerbation of symptoms resulted in doubling of the length of stay in hospital (from 18 to 33 days). Chebli et al. found a similar number of days of abdominal pain before oral refeeding in those that did and did not relapse [75]. Pain relapse was predicted by peripancreatic fluid collection, serum CRP on the 4th day, and serum lipase on the day of oral feeding.

Jacobson et al. hypothesized that patients recovering from mild AP would be discharged from the hospital sooner if they resumed oral nutrition with a low-fat solid diet compared with a clear liquid diet [67]. Patients with mild pancreatitis were randomized to a clear liquid diet or low-fat solid diet when they were ready to resume oral nutrition. Patients were monitored daily for recurrence of pain, need to stop feeding, post-refeeding length of hospital stay (primary endpoint), and for 28 days post-refeeding to capture readmission rates. 1335 patients were assessed for eligibility and 66 allocated to a clear liquid diet (588 kcal, 2 g fat) and 55 to a low fat solid diet (1200 kcal, 35 g fat). Because of the large number of excluded patients, a bias by selection may have occurred. The number of patients requiring cessation of feeding because of pain or nausea was similar (6% and 11%, resp.), the median length of stay after refeeding was similar, and there was no difference in the 28-day readmission rates. Patients on the low fat diet consumed significantly more calories and grams of fat during their first meal and on study day 1 (301 kcal and 2 g fat versus 622 kcal and 13 g fat, *P* < .001). Initiating oral nutrition after mild pancreatitis with a low fat solid diet appeared to be safe and provided more calories than a clear liquid diet, but did not result in a shorter length of hospitalization. The abdominal pain score on the day of refeeding was associated with a failure of oral intake with those experiencing more pain having a higher likelihood of being made nil per mouth. Unfortunately, the authors failed to resolve the important question of what the optimal diet should be in patients recovering from mild pancreatitis. Sathiaraj et al. performed a randomized trial to determine the length of hospital stay and tolerance to oral refeeding in patients with mild AP and acute on chronic pancreatitis when started on a soft diet (*n* = 52, 1040 kcal and 20 g fat) as compared to a clear liquid diet (*n* = 49, 458 kcal and 11 g fat) [68]. The length of hospital stay (post-refeeding and total) decreased significantly on a soft diet. They observed no significant difference in the need for cessation of feeding because of pain or nausea. Patients on the soft diet consumed significantly more calories and grams of fat during their first meal and on study day 1 (921 kcal and 15 g fat versus 370 kcal and 8 g fat, *P* < .001). They concluded that oral refeeding with a soft diet was safe and resulted in a shorter length of hospital stay. However, in both nonblinded studies, a definition of when to discharge patients was not given. Hospital discharge was decided by the medical team without input from study team.

## 8. Guidelines of Enteral Nutrition in Acute Pancreatitis

Recently, several general practice guidelines for AP have been published [1, 4, 8, 76–78]. These comment on nutritional management in mild and severe AP. The European Society of Parenteral and Enteral Nutrition (ESPEN) published a revised and comprehensive guideline on EN in AP in 2006 [8]. The several guidelines cover mostly the same recommendations. To date some of the recommendations require updating according to the best available evidence as discussed above. Generally, for mild AP it is recommend to initiate EN if patients cannot consume normal food after 5–7 days. For severe AP nutritional support is indicated when it becomes evident that the patient will not be able to tolerate oral intake for a prolonged period of time, for example, for at least 7 days. This assessment can usually be made within the first 3-4 days of admission. EN should be supplemented by parenteral nutrition if needed. Also, in severe pancreatitis with complications such as pancreatic fistulas, ascites, and pseudocysts, tube feeding can be given uneventfully. If gastric feeding is not tolerated, the jejunal route should be tried and continuous feeding in stead of bolus feeding should be used. In gastric outlet obstruction, feeding beyond the obstruction with the tube tip distal to the obstruction should be tried. If this is impossible, parenteral nutrition should be given. In case of surgery for complications of AP, an intraoperative jejunostomy for postoperative feeding is feasible. 

Peptid-based semielemental formulas can be used safely and standard formulae can be tried if they are tolerated.

## 9. Summary of Recent Developments

Most patients with AP have mild disease and do not need additional nutritional support during admission. According to the guidelines, nutritional support is indicated if patients cannot consume normal food after 5–7 days or when it becomes evident that the patient will not be able to tolerate oral intake for a prolonged period of time (7 days or more). When artificial nutrition is indicated, EN is preferred over TPN, because it reduces complications and mortality in AP when compared with TPN. TPN should only be used in patients unable to tolerate EN. It is likely that EN has a beneficial influence on the disease course and should be initiated as early as possible (within 48 hours of admission). With some caution it can be stated that nasogastric tube feeding in severe AP is possible, making the concept of pancreatic rest less probable. However, larger multicentre studies are needed to confirm the safety and effectiveness of nasogastric feeding when compared to nasojejunal feeding and to investigate the role of early (within 48 hours) versus late nutrition support. Randomized controlled trails have been started and will hopefully give a more definite answer. The clinical evidence for the use of just (semi) elemental formulas is weak. Supplementation of enteral formulas with glutamine and prebiotics and the use of immune enhancing formulas cannot routinely be recommended. Probiotics should not be administered routinely in patients with predicted severe AP. To date, some of the recommendations as stated in the latest guidelines require updating according to the best available evidence. 

## Abbreviations

AP: Acute pancreatitis
NPO: Nil per os
EN: Enteral nutrition
TPN: Total parenteral nutrition
SIRS: Systemic inflammatory response syndrome
MOF: Multiorgan failure
ICU: Intensive care unit
RCT: Randomized controlled trial
CCK: Cholecystokin
VIP: Vasoactive intestinal peptide.
